# EHMTI-0252. The frequency of acute medication intake relates to its perceived effectiveness in chronic headache patients

**DOI:** 10.1186/1129-2377-15-S1-E23

**Published:** 2014-09-18

**Authors:** DML Van Ryckeghem, T Wens, K Paemeleire, G Crombez

**Affiliations:** 1 Department of Experimental Clinical and Health Psychology, Ghent University, Ghent, Belgium; 2 Department of Neurology, University Hospital Ghent, Ghent, Belgium

## Introduction

Although the benefit of acute pain medications for chronic headaches (≥ 15 days/month) often is limited, patients frequently continue to take them in an attempt to control their pain, which may then result in medication overuse.

## Aims

The current study investigates whether the intake of acute medication creates an illusion of control (IoC) and whether an accepting attitude may prevent this IoC in chronic headache patients.

## Method

Forty-one chronic headache patients (primarily migraine sufferers) filled out a questionnaire battery (e.g. CPAQ and MIDAS) followed by an adapted IoC Task. During this IoC task, patients were asked on each trial to indicate whether they would (fictively) take a new painkiller to cope with a (fictive) headache day. Afterwards the patient received random information on the effectiveness of the painkiller that day. After the task participants filled out 6 questions on the perceived effectiveness of this painkiller.

## Results

Data show that the perceived effectiveness was positively related to the number of times patients have fictively taken the painkiller (p<.01) and not to its actual contingency (i.e., actual effectiveness). Secondly, acceptance was positively related to perceived effectiveness of the painkiller (p<.05), but did not moderate the relationship between perceived effectiveness and frequency of medication intake.

## Conclusions

Results of the present study suggest that a higher intake of acute medication by chronic headache patients is related to higher perceived effectiveness of the acute medication, independent of its actual effectiveness. This IoC-effect is however not influenced by acceptance.

No conflict of interest.

